# Supplementary material for: Respiration as a dynamic modulator of sensory sampling
Source: Nat Commun. 2026 Apr 7;17:3261. doi: 10.1038/s41467-026-71604-8 (PMC13062087; doi:10.1038/s41467-026-71604-8)
Supplement: Supplementary file 1 — Supplementary Information [file 41467_2026_71604_MOESM1_ESM.pdf]

Supplementary Information

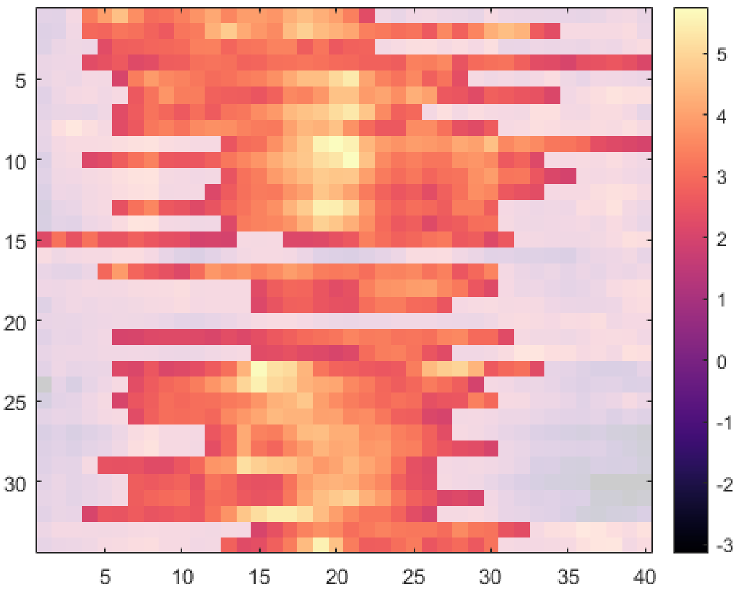

Supplementary Figure S1. Power spectra for each ROI without removal of the 1/f component through spectral whitening.

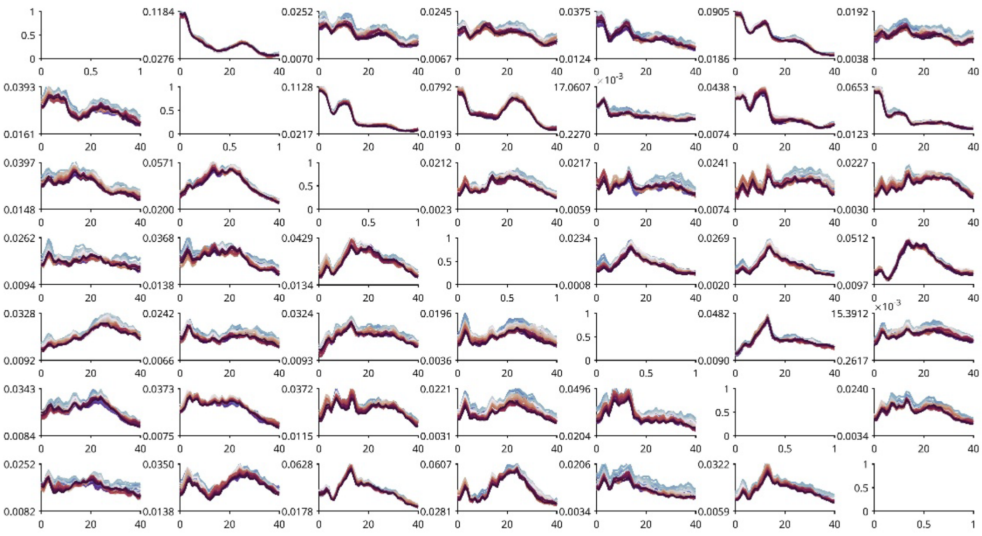

Supplementary Figure S2. DAI spectra as a function of respiratory phase for each combination of ROIs.

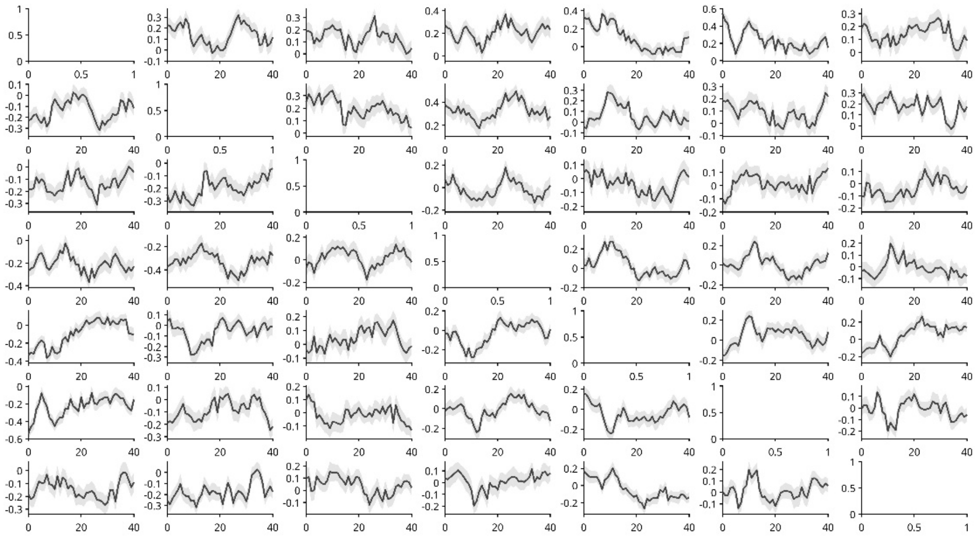

Supplementary Figure S3. DAI spectra of regression weights (Granger causality as a function of respiration phase) for each combination of ROIs.
