# Supplementary material for: Respiration as a dynamic modulator of sensory sampling
Source: Nat Commun. 2026 Apr 7;17:3261. doi: 10.1038/s41467-026-71604-8 (PMC13062087; doi:10.1038/s41467-026-71604-8)
Supplement: Supplementary file 2 — Reporting Summary [file 41467_2026_71604_MOESM2_ESM.pdf]

nature portfolio

Corresponding author(s): Daniel Kluger

Last updated by author(s): Feb 5, 2026

Reporting Summary

Nature Portfolio wishes to improve the reproducibility of the work that we publish. This form provides structure for consistency and transparency in reporting. For further information on Nature Portfolio policies, see our Editorial Policies and the Editorial Policy Checklist.

Statistics

For all statistical analyses, confirm that the following items are present in the figure legend, table legend, main text, or Methods section.

n/a	Confirmed	
☐	☒	The exact sample size (*n*) for each experimental group/condition, given as a discrete number and unit of measurement
☐	☒	A statement on whether measurements were taken from distinct samples or whether the same sample was measured repeatedly
☐	☒	The statistical test(s) used AND whether they are one- or two-sided<br>*Only common tests should be described solely by name; describe more complex techniques in the Methods section.*
☐	☒	A description of all covariates tested
☐	☒	A description of any assumptions or corrections, such as tests of normality and adjustment for multiple comparisons
☐	☒	A full description of the statistical parameters including central tendency (e.g. means) or other basic estimates (e.g. regression coefficient) AND variation (e.g. standard deviation) or associated estimates of uncertainty (e.g. confidence intervals)
☐	☒	For null hypothesis testing, the test statistic (e.g. *F*, *t*, *r*) with confidence intervals, effect sizes, degrees of freedom and *P* value noted<br>*Give P values as exact values whenever suitable.*
☒	☐	For Bayesian analysis, information on the choice of priors and Markov chain Monte Carlo settings
☒	☐	For hierarchical and complex designs, identification of the appropriate level for tests and full reporting of outcomes
☐	☒	Estimates of effect sizes (e.g. Cohen's *d*, Pearson's *r*), indicating how they were calculated

Our web collection on statistics for biologists contains articles on many of the points above.

Software and code

Policy information about availability of computer code

Data collection	No software was used for data collection
Data analysis	All custom Matlab code to reproduce the central findings of this study will be made openly available from the Open Science Framework upon publication.<br>Fieldtrip (version 20240111) for Matlab 2024b, circstat toolbox, watsons_U2 function for Matlab

For manuscripts utilizing custom algorithms or software that are central to the research but not yet described in published literature, software must be made available to editors and reviewers. We strongly encourage code deposition in a community repository (e.g. GitHub). See the Nature Portfolio guidelines for submitting code & software for further information.

Data

Policy information about availability of data

All manuscripts must include a data availability statement. This statement should provide the following information, where applicable:

- Accession codes, unique identifiers, or web links for publicly available datasets
- A description of any restrictions on data availability
- For clinical datasets or third party data, please ensure that the statement adheres to our policy

The processed and anonymised data generated in this study are publicly available from the Open Science Framework via osf.io/qasvp

Human research participants

Policy information about studies involving human research participants and Sex and Gender in Research.

Reporting on sex and gender	No hypotheses regarding the effects of sex or gender were included in the study. Information regarding participants' sex and gender were acquired by means of self report and balanced across the sample.
Population characteristics	Thirty right-handed volunteers (15 female, age 25.9 ± 3.3 y [mean ± SD]) participated in the study. All participants reported having no respiratory or neurological disease and gave written informed consent prior to all experimental procedures.
Recruitment	Participants were recruited from the University's data base. The responsible lab manager was otherwise not involved in conducting the studies, effectively avoiding selection bias during participant acquisition.
Ethics oversight	The study was approved by the local ethics committee of the University of Münster (Medical Faculty, ID 2018-068-f-S).

Note that full information on the approval of the study protocol must also be provided in the manuscript.

Field-specific reporting

Please select the one below that is the best fit for your research. If you are not sure, read the appropriate sections before making your selection.

[x] Life sciences   [ ] Behavioural & social sciences   [ ] Ecological, evolutionary & environmental sciences

For a reference copy of the document with all sections, see nature.com/documents/nr-reporting-summary-flat.pdf

Life sciences study design

All studies must disclose on these points even when the disclosure is negative.

Sample size	The sample sizes at each individual recording lab were determined based on prior studies on respiration-brain coupling (Kluger & Gross, 2020; Kluger & Gross, 2021; Kluger et al., 2021).
Data exclusions	No data were excluded from the analyses.
Replication	N/A
Randomization	Trials and conditions were randomized across participants.
Blinding	As our experimental design did not include between-participant or between-session variables, blinding was not necessary.

Reporting for specific materials, systems and methods

We require information from authors about some types of materials, experimental systems and methods used in many studies. Here, indicate whether each material, system or method listed is relevant to your study. If you are not sure if a list item applies to your research, read the appropriate section before selecting a response.

Materials & experimental systems

n/a	Involved in the study	
[x]	[ ]	Antibodies
[x]	[ ]	Eukaryotic cell lines
[x]	[ ]	Palaeontology and archaeology
[x]	[ ]	Animals and other organisms
[x]	[ ]	Clinical data
[x]	[ ]	Dual use research of concern

Methods

n/a	Involved in the study	
[x]	[ ]	ChIP-seq
[x]	[ ]	Flow cytometry
[ ]	[x]	MRI-based neuroimaging

Magnetic resonance imaging

Experimental design

Design type	Block design
Design specifications	Single standard T1-weighted sequence for the acquisition of structural data.

Behavioral performance measures	None

Acquisition

Imaging type(s)	Structural
Field strength	3T
Sequence & imaging parameters	Anatomical images were acquired using a standard Siemens 3D T1-weighted whole brain MPRAGE imaging sequence (1 x 1 x 1 mm voxel size, TR = 2130 ms, TE = 3.51 ms, 256 x 256 mm field of view, 192 sagittal slices).
Area of acquisition	Whole-brain

Diffusion MRI ☐ Used ☐ Not used

Preprocessing

Preprocessing software	Fieldtrip and SPM12
Normalization	Co-registration of structural MRIs to the MEG coordinate system was done individually by initial identification of three anatomical landmarks (nasion, left and right pre-auricular points) in the participant's MRI. Using the implemented segmentation algorithms in Fieldtrip and SPM12, individual head models were constructed from anatomical MRIs. A solution of the forward model was computed using the realistically shaped single-shell volume conductor model with a 5 mm grid defined in the MNI template brain (Montreal Neurological Institute, Montreal, Canada) after linear transformation to the individual MRI.
Normalization template	MNI305
Noise and artifact removal	T1 images were corrected for field inhomogeneities using SPM12
Volume censoring	T1 images were motion-corrected using SPM12

Statistical modeling & inference

Model type and settings	Linear regression model predicting Granger causality and DAI by fixed effects for respiration phase (vector norm of respiratory sine and cosine).
Effect(s) tested	Granger causality and DAI across respiration phase

Specify type of analysis: ☐ Whole brain ☒ ROI-based ☐ Both

Anatomical location(s): The posterior ROI was derived from a previous study (Kluger et al., 2021).

Statistic type for inference (See Eklund et al. 2016)	Not applicable due to averaging within the ROI
Correction	Permutation testing

Models & analysis

n/a	Involved in the study
☐	☒ Functional and/or effective connectivity
☒	☐ Graph analysis
☒	☐ Multivariate modeling or predictive analysis

Functional and/or effective connectivity	We used a non-parametric approach to estimate pairwise multivariate Granger causality between each pair of ROIs. The spectral representation of the source-localised time series was estimated using the fast Fourier transform along with multitapers (using 2-Hz smoothing) on the time domain data (separately for each condition) for the time interval of [-1600 0] ms relative to target onset. For each pair of ROIs we computed the cross-spectral density matrix and used it to compute multivariate Granger Causality (mGC) using a nonparametric spectral matrix factorization in a blockwise approach. For this, we considered the first three principal components of each ROI's source-localised, trial-based time series.
